# Proteome Damage Inflicted by Ionizing Radiation: Advancing a Theme in the Research of Miroslav Radman

**DOI:** 10.3390/cells10040954

**Published:** 2021-04-20

**Authors:** Steven T. Bruckbauer, Benjamin B. Minkoff, Michael R. Sussman, Michael M. Cox

**Affiliations:** 1 Department of Biochemistry, University of Wisconsin-Madison, Madison, WI 53706, USA; steven.bruckbauer@gmail.com (S.T.B.); msussman@wisc.edu (M.R.S.); 2 Center for Genomic Science Innovation, University of Wisconsin-Madison, Madison, WI 53706, USA; bminkoff@wisc.edu

**Keywords:** proteome, *Escherichia coli*, *Deinococcus radiodurans*, reactive oxygen species, protein damage, ionizing radiation

## Abstract

Oxidative proteome damage has been implicated as a major contributor to cell death and aging. Protein damage and aging has been a particular theme of the recent research of Miroslav Radman. However, the study of how cellular proteins are damaged by oxidative processes is still in its infancy. Here we examine oxidative changes in the proteomes of four bacterial populations—wild type *E. coli*, two isolates from *E. coli* populations evolved for high levels of ionizing radiation (IR) resistance, and *D. radiodurans*—immediately following exposure to 3000 Gy of ionizing radiation. By a substantial margin, the most prominent intracellular oxidation events involve hydroxylation of methionine residues. Significant but much less frequent are carbonylation events on tyrosine and dioxidation events on tryptophan. A few proteins are exquisitely sensitive to targeted oxidation events, notably the active site of glyceraldehyde 3-phosphate dehydrogenase (GAPDH) in *E. coli*. Extensive experimental evolution of *E. coli* for IR resistance has decreased overall proteome sensitivity to oxidation but not to the level seen in *D. radiodurans*. Many observed oxidation events may reflect aspects of protein structure and/or exposure of protein surfaces to water. Proteins such as GAPDH and possibly Ef-Tu may have an evolved sensitivity to oxidation by H_2_O_2_.

## 1. Introduction

Cells growing aerobically must deal with reactive oxygen species (ROS) as byproducts of respiration. These include (proceeding from most oxidized to most reduced) superoxide radicals, hydrogen peroxide, and hydroxyl radicals. These exhibit half-lives of ~5 s, days to weeks, and ~10^–9^ s, respectively. The highly reactive and very short-lived hydroxyl radicals damage anything in their immediate vicinity. Enzymes exist in most cells to eliminate superoxide radicals (superoxide dismutase) and peroxides (glutathione peroxidase and catalases), but not the short-lived hydroxyl radicals. Conversion of the radical species to longer lived and diffusible H_2_O_2_ can lead to oxidation of sulfydryls and other groups, and also participate in the Fenton reaction with FeII. Fe-S clusters, with their important redox, catalytic, and regulatory functions, may be significant targets, as well as mononuclear iron proteins [1,2]. The Fenton reaction would not simply damage the Fe-S clusters but would also generate more hydroxyl radicals. These and related processes slowly damage cellular proteins and peptides and may contribute to aging in higher eukaryotes [3,4,5,6,7,8,9,10]. The connection between protein oxidation and aging has been a theme in the recent research from the laboratory of Miroslav Radman [11,12,13,14,15], to which this issue and article are dedicated. The role of protein oxidation in aging remains an important focus of research [3,4,5,6,7,8,9,10].

Many questions remain to be answered about proteome damage via these oxidative processes. How fast does it occur? What proteins and parts of proteins are most susceptible to damage? How much of that damage can be repaired? There are many more. The effects of ionizing radiation (IR) on cellular proteomes can provide some important clues. IR such as gamma radiation (X-rays, ^60^Co decay, etc.) causes damage primarily via radiolysis of water and dissolved O_2_, generating ROS. High doses of IR can be administered in short amounts of time allowing investigators to rapidly increase intracellular levels of ROS and examine the effects of such an extreme stress.

In wild type *E. coli*, an IR dose of 1000 Gy kills most of the cells. The proteome suffers relatively little damage [16] but the genome is fragmented by IR-induced double-strand breaks (DSBs) [16]. Cell death is most likely due to the DNA damage caused by ROS, including hydroxyl radicals from various sources including Fenton chemistry. However, damage to other cellular components, such as Fe-S complexes (also via Fenton chemistry) may play a role [1,2]. Additional oxidation events may cause enzyme inactivation and cell wall damage.

Using experimental evolution, we have been pursuing a long-term project to generate populations of *Escherichia coli* that are as radiation resistant as the bacterium *Deinococcus radiodurans* [17,18,19,20]. Progress reports have been published [17,18]. IR resistance has continued to increase. In addition to the evolution experiment, we have begun to catalogue proteome damage imposed by exposure to IR in both wildtype *E. coli* and these evolved isolates. We previously focused on wild type *E. coli* and *D. radiodurans*, examining proteome damage inflicted by 1000 Gy. Whereas this dose is sufficient to kill more than 99.9% of *E. coli* cells, it does not result in measurable lethality in *D. radiodurans* [16]. Limited proteome damage was noted in *E. coli* (only 22 of 13,262 peptides detected were both oxidized and exhibited a fold increase in abundance >2) with little to no damage seen in *D. radiodurans*. One major oxidation event, trioxidation of an active site Cys residue in glyceraldehyde 3-phosphate dehydrogenase (GAPDH), dominated the oxidative spectrum but was not present in *Deinococcus* [16]. In the present report, we examine proteome damage inflicted by much higher doses of IR. We compare *Escherichia coli* and *Deinococcus radiodurans*, as well as two isolates from highly evolved populations generated in the evolution trials [17,18]. Our goal was to continue cataloguing the types of protein oxidation events that occur as well as to begin to determine the extent to which IR-resistance is contingent on a capacity for amelioration of protein oxidation comparable to *Deinococcus*. Using a dose of 3000 Gy, an increased level of damage is evident, allowing a more systematic examination of oxidative damage to bacterial proteomes.

## 2. Materials and Methods

### 2.1. Growth Conditions and Bacterial Strains Used in This Study

Unless otherwise stated, *E. coli* cultures were grown in Luria–Bertani (LB) broth [21] at 37 °C with aeration. *E. coli* were plated on 1.5% LB agar medium [21] and incubated at 37 °C. Overnight cultures were grown in a volume of 3 mL for 16 to 18 h. Exponential phase cultures were routinely diluted 1:100 in 10 mL of LB medium in a 50 mL Erlenmeyer flask and were grown at 37 °C with shaking at 200 rpm and were harvested at an OD_600_ of 0.2, unless otherwise noted. After growth to an OD_600_ of 0.2, cultures were placed on ice for 10 min to stop growth before being used for assays.

All *E. coli* strains used are derivatives of *E. coli* K-12 derivative MG1655 [22], two of which (IR9-150-1 and IR9-150-2) were generated through 150 cycles of selection with ionizing radiation [17,18]. *D. radiodurans* is an R1 derivative (ATCC13939) [23]. Overnight liquid cultures were incubated at 30 °C with shaking for 24 h; exponential phase cultures were prepared by 1:100 dilution of overnight cultures into 10 mL of 2X TGY medium [24] and incubated at 30 °C with shaking to an OD_600_ of 0.08–0.16.

All experiments were performed using biological replicates of cultures generated from isolated colonies.

### 2.2. Irradiation of Bacterial Cultures Using the Linac+

Strains were grown in biological quintuplicate overnight, diluted 1:100 into 50 mL of fresh LB medium, and outgrown to an OD_600_ of approximately 0.2 in LB as routinely performed (or in 2X TGY to an OD_600_ of 0.08–0.16 for *D. radiodurans*). A 40 mL aliquot of each early exponential phase culture was pelleted by centrifugation at 3500 rpm (2575× *g*) for 10 min at 4 °C. Supernatants were poured off, and samples resuspended in 40 mL of ice-cold 1X phosphate-buffered saline (PBS) (for 1 L: 8 g NaCl, 0.2 g KCl, 1.44 g Na_2_HPO_4_, KH_2_PO_4_ 0.24 g with 800 mL dH_2_O, adjust pH with HCl to 7.4, then add remaining 200 mL dH_2_O) and pelleting was repeated. This process was repeated twice more with cells resuspended in 20 mL ice-cold PBS, and a final time suspending in 500 µL ice-cold 1X PBS. Two 100 µL aliquots were made for each culture in 1.5 mL microfuge tubes for mass spectrometry analysis of cells receiving 0 or 3000 Gy of IR from the Linac.

### 2.3. Generalized Linac Irradiation Protocol

Irradiations were performed as previously described [16]. Samples were maintained at 4 °C and transported to the University of Wisconsin Medical Radiation Research Center (UWMRRC) Varian 21EX clinical linear accelerator (Linac) facility for irradiation. The total transport time was approximately 15 min to and from the Linac facility. For each irradiation, the Linac was set to deliver a beam of electrons with 6 MeV of energy to uniformly irradiate all samples (a total of 28) at once. To accomplish this, a special high-dose mode called HDTSe^−^ was utilized, which resulted in a dose rate to the samples of approximately 72 Gy/min. The sample tubes were placed horizontally and submerged at a depth of 1.3 cm (measured to the center of the tube’s volume) in an ice-water filled plastic tank and set to a source-to-surface distance (SSD) of 61.7 cm. A 30 by 30 cm^2^ square field size was set at the Linac console, which gave an effective field size at this SSD of 18.5 by 18.5 cm^2^. This is ample coverage to provide a uniform dose to all of the sample vials. The monitor unit calculations (determination of the amount of time to leave the Linac on) were based on the American Association of Physicists in Medicine (AAPM) Task Group 51 protocol for reference dosimetry [25]. This is the standard method for determining dose per monitor unit in water for radiation therapy calculations. Once the dose was determined in the AAPM Task Group 51 reference protocol conditions (SSD = 100 cm and depth = 10 cm), an ion chamber and water-equivalent plastic slabs were used to translate this dose to the specific conditions used in this project. An independent dose verification was performed with thermoluminescent dosimeters (TLDs) [17]. TLDs are passive dosimeters that are small, accurate and well-suited for dose verification in the routinely used 1.5 mL sample vials.

### 2.4. Label Free Quantification Mass Spectrometry with E. coli and D. radiodurans

Irradiated (3000 Gy) and untreated (0 Gy) samples were lysed by addition of SDS to a final concentration of 2.0%, then immediately subjected to protein extraction and concentration using a standard methanol:chloroform protocol. Purified protein pellets were solubilized in 8 M urea with 50 mM ammonium bicarbonate (AMBIC) and subjected to a standard BCA assay to determine protein concentration.

For each of the samples, 10 µg (varying volumes) of each was diluted to 4 M urea with 50 mM AMBIC and treated with 2 mM dithiothreitol for 30 min at 50 °C, 5 mM iodoacetamide for 30 min at room temperature in darkness, and then 2 mM dithiothreitol for 5 min at room temperature. Samples were diluted further to 1 M urea with 50 mM AMBIC, and 0.05 µg of trypsin and Lys-C proteases were each added (final protease mass:protein mass of 1:100). Samples were incubated overnight at 37 °C, for 15 h total.

Digestions were stopped with addition of neat formic acid to 1.0%, subjected to solid phase cleanup using Agilent C18 OMIX tips (Agilent Technologies; Santa Clara, CA, USA) according to manufacturer’s protocol, and dried down to completion using a vacuum centrifuge.

Samples were injected for analysis using an UltiMate 3000 RSLCnano system (Thermo Fisher; Waltham, MA, USA) onto an Orbitrap Lumos mass spectrometer (Thermo Fisher; Waltham, MA, USA). A 75 µm by 50 cm Pepmap RSLC column (Thermo Fisher; Waltham, MA) packed with 2 µm beads and 100 Å pore size was used as the stationary phase. Mobile phase A was 0.1% formic acid and mobile phase B was 80% acetonitrile/0.1% formic acid. A 75-min elution gradient to 37.5% B was used, after which 95% B was flushed for 5 min and column re-equilibration using 2% B was performed for 10 min. DDA-MS was performed with the following parameters: MS1 spectra were acquired in profile mode in the Orbitrap with a resolution of 120K and a scan range of 350–1600 *m*/*z*. A normalized AGC target of 250% and automatic max inject time was used. Charge state filtering of 2–7, monoisotopic peak selection set to peptide, and dynamic exclusion of 10 s, *n* = 1 and with a mass tolerance of +/−10 ppm were used for triggering MS2 acquisition. Cycle time between MS1 scans was set to a max of 1 s. For MS2 acquisition, an isolation window of 0.7 Da was used and peptides were fragmented using HCD with a collision energy of 32%. MS2 were acquired in centroid mode in the Ion Trap using the automatic scan range parameter and scan rate set to turbo. An AGC target of 3e4 and an automatic max inject time were used.

Data was analyzed using the Sequest algorithm within Proteome Discoverer (PD) (Thermo Fisher; Waltham, MA, USA). For *E. coli* searches, the Uniprot K12 *E. coli* proteome, downloaded on 7 February 2019, was used (PID: UP000000625, 4382 sequences including contaminants). For *D. radiodurans* searches, the Uniprot *D. radiodurans* proteome, downloaded on 3/27/2018, was used (PID: UP000002524, 3172 sequences including contaminants). Databases were searched with the specified parameters: trypsin with 2 possible missed cleavages, precursor and fragment mass tolerance 10 ppm and 0.6 Da, respectively, and a max amount of 4 dynamic modifications per peptide. Dynamic modifications were specified as carbamidomethyl/+57.021 Da (on C), oxidation/+15.995 Da (on CDEFHILMNPQRSTVWY), carbonylation/+13.979 Da (on ACDEFHILKMNPQRSTVWY), dioxidation/+31.990Da (on ACDEFHIKLMNPRSTVWY), and trioxidation/+47.985 Da (on CFWY). No static modifications were set. Searches were based on previous reports of abundance of the given modifications on each amino acid reside [26]. A false discovery rate (FDR) for peptide spectral matches (PSMs), peptides, and proteins of 0.05% was used via percolator in PD. For quantification, a combination of the Minora Feature Detector, Feature Mapper, and Precursor Ions Quantifier nodes were used in PD. Samples were grouped per strain or organism as 5 untreated replicates untreated vs. 5 treated replicates. Default settings were used for the Minora Feature Detector and Feature Mapper nodes. For the Precursor Ion Quantifier node, Intensity was used for precursor quantification and normalization was performed using total peptide amount per file. Both unique and razor peptides were used to quantify protein level differences, excluding modified peptides. Protein levels were quantified using summed abundances, no data imputation, and ANOVA hypothesis testing on individual proteins. The Benjamini–Hochberg method was used to adjust *p* values, and the adjusted *p* values that were used for significance thresholds.

## 3. Results

Previous work has often examined biological responses to IR exposure. Here, we wish to quantify the immediate, abiotic chemical effects of IR on the in vivo proteome, prior to any biological response. To ensure our observations of IR-induced changes to the *Escherichia coli* proteome are indeed abiotic, cultures were cooled to 4 °C prior to irradiation. Unlike the previous study, *E. coli* cells were grown in LB rich media throughout irradiation, due to diminished growth rate of the evolved isolates in media other than LB. However, all cells were extensively washed in 1X PBS to halt metabolism and remove nutrients in growth media that may act as a radioprotectant. In addition, sample tubes were submerged in cold (4–10 °C) water throughout irradiation. Samples were frozen in liquid nitrogen immediately after irradiation and remained frozen and protected from light and O_2_ until processing. In this study, IR was delivered at 70 Gy/min by a high energy electron beam linear accelerator (Linac) commonly used in cancer radiotherapy. Under the conditions used in our experiments, a dose of 3000 Gy was administered quickly (~40 min). Based on previous measurements, this dose is sufficient to cause approximately 60 DNA DSBs per cell, and is also expected to induce significant oxidative damage to proteins [27].

In order to provide several comparative proteomes from IR-resistant species, we carried out identical experiments as laid out above with (a) two isolates from a population that had been subjected to 150 rounds of selection for IR resistance as part of a long-term evolution trial to generate high levels of IR resistance [17,18] and (b) the highly radioresistant bacterium *Deinococcus radiodurans*. The isolates from the experimental evolution trial are both from one population (IR9) and are denoted IR9-150-1 and IR9-150-2. They are from two different sub-populations within IR9 that are involved in persistent clonal interference [18]. The ability of *Deinococcus* to protect its proteome from IR-generated ROS is a well-studied phenomenon [27,28,29,30]. At a dose of 3000 Gy, this bacterium exhibits no lethality when subjected to a slower dose rate of IR up to 5000 Gy [30,31,32,33,34,35]. However, a dose of 3000 Gy administered from the Linac kills 50–90% of the cells in a culture of *D. radiodurans* [17,18]. Survival of the *E. coli* isolates subjected to experimental evolution is approaching that of *D. radiodurans* [17,18]. The dose of 3000 Gy was chosen to provide a broader spectrum of proteome damage than that catalogued in an earlier study [16].

Samples were thawed and processed immediately. To survey and quantify as much IR-induced oxidation as possible, peptides from ten replicates (five treated and five mock-treated) were analyzed using mass spectrometry (MS). The proteome databases were searched systematically for previously identified IR-induced amino acid modifications, including carbonylation (+13.98 Da), hydroxylation (+15.99 Da), dioxidation (+31.99 Da), and trioxidation (+47.99 Da) [26,36]. We note here that although we classify +31.99 Da as dioxidation, such a mass shift could also be peroxidation. Though these modifications represent different chemistries, we cannot distinguish between the two in these data. We also note that we are using general terms for modification here, though more specific terminology will be used in some instances (i.e., +47.99 Da on Cys results in a sulfonic acid chemical group, +13.98 Da on Pro can represent conversion to pyroglutamic acid, etc.). Given their scarcity in previous work, we did not search for additional previously reported but less-common modifications [26,36,37].

The coverage in our datasets is summarized in Table 1, Table 2, Table 3 and Table 4. The total number of peptides detected ranged from 14,738 to 21,516 in the samples obtained from the three *E. coli* strains and 14,440 from *D. radiodurans*, respectively. Unique proteins detected in each strain were: MG1655, 2020; IR9-150-1, 1868; IR9-150-2, 1703; *D. radiodurans*, 954. This corresponded to nearly half of the predicted proteome for each strain, or most of the proteins typically expressed during exponential phase growth [38]. Of the peptides detected, a relatively small fraction contained oxidative modifications (Table 1, Table 2, Table 3 and Table 4). The highlighting added to draw attention to the relative occurrence of major oxidation classes.

**Figure 1:**
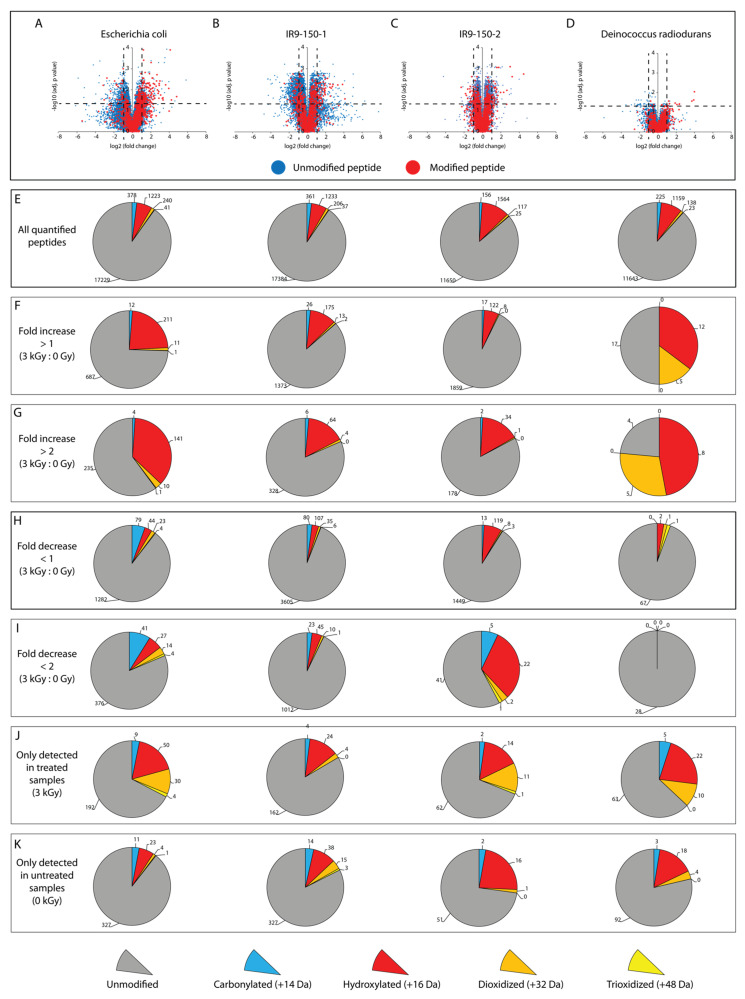
Spectrum of peptide oxidation of cultures irradiated with 3 kGy. (**A**) volcano plots depict the fold increase or decrease (log2 transformed) and statistical significance of the fold change (−log10 transformed Benjamini–Hochburg adjusted *p*-value) of peptides sequenced using label free quantification mass spectrometry (LFQ-MS) of biological quintuplicate samples of 3 kGy treated vs. 0 Gy treated cultures of *E. coli* MG1655, experimentally evolved ionizing radiation (IR)-resistant *E. coli* isolates (**B**) IR9-150-1 and (**C**) IR9-150-2, and radioresistant bacterium (**D**) *Deinococcus radiodurans*. Each dot represents a single peptide, where unmodified peptides are blue and oxidized peptides are red. Dashed lines indicate significance thresholds (fold change greater than 2, adjusted *p*-value less than 0.05). Pie charts indicate the raw number of peptides that are unmodified (gray), carbonylated (blue), hydroxylated (red), dioxidized (orange), and trioxidized (yellow) at various thresholds: (**E**) all quantified peptides, (**F**) peptides with a significant increase greater than 1, (**G**) peptides with a significant increase greater than 2, (**H**) peptides with a significant decrease greater than 1, (**I**) peptides with a significant decrease greater than 2, (**J**) peptides detected only in the irradiated samples, and (**K**) peptides detected only in the unirradiated samples. In all four strains, most peptides detected are unmodified, and the most prevalent type of oxidation is hydroxylation (+16 Da). Both evolved isolates (IR9-150-1 and IR9-150-2) and *D. radiodurans* exhibit fewer oxidized peptides with increased abundance compared to *E. coli* MG1655, suggestive of an ability to suppress IR-induced protein oxidation.

We tabulated the peptides that exhibited either changes or no change in abundance in the irradiated samples relative to the unirradiated samples, focusing first on the vast majority that exhibited no statistically significant change in abundance after IR treatment at 3000 Gy (Table 1, Table 2, Table 3 and Table 4). Most of these peptides were unmodified. The overall patterns of detected peptides are shown in Figure 1.

A subset of the peptides that exhibited no changes in abundance were modified. There are two potential sources contributing to a background of oxidatively modified peptides that do not change in abundance due to IR exposure, endogenous intracellular ROS species or source oxidation during electrospray ionization. Since we froze the samples in this study immediately after irradiation and protected them from the environment, the involvement of endogenous ROS should be reduced relative to a previous study [16]. The observed background of oxidized peptides is similar in both *E. coli* and *D. radiodurans*. *D. radiodurans* has a documented enhanced capacity to ameliorate ROS [29,39]. If we assume that *D. radiodurans* is better able to suppress intracellular ROS than is *E. coli*, yet the background of oxidatively modified peptides is similar or even higher than in *E. coli*, the likely origin of our oxidized peptide background is in-source oxidation during electrospray ionization of peptides. Determining the precise source of these IR-independent modifications is a continuing effort but is outside the focus of this study. The background modification is in our whole cell samples and is consistent with previous observations of endogenous ROS produced during growth in nutrient-rich medium [40,41] and oxidation of proteins during the electrospray ionization step of MS [42]. For the subset of oxidized peptides that showed no change in abundance, single hydroxylation (+15.99 Da), especially on methionine, was the most prevalent modification (~7–12%) (Table 5).

The other remaining peptides exhibited a statistically significant increase or decrease in abundance (fold change greater than 2, adjusted *p*-value < 0.05) (Table 1, Table 2, Table 3 and Table 4). Again, most of these peptides were unmodified. In principle, changes in abundance of unmodified peptides can occur in at least three ways. There could be an increase in synthesis, increase or decrease in proteolysis, both increase in synthesis and a change in proteolysis. Changes to proteolysis would reflect changes in access to trypsin cleavage sites. The extraction procedure, which involves the use of detergents, should eliminate most protein–protein interactions, so changes in association should not unduly affect our access to peptides. However, in principle, any interactions not eliminated by our protocol might be affected instead by the irradiation, leading to increased access to trypsin cleavage sites and an observed increase in certain peptides. Our attempts to suppress cell metabolism during irradiation may not be perfect. In spite of the absence of growth and nutrients, as well as the cold temperature to halt metabolism, certain proteins may increase in abundance during the 40 min irradiation process. Alternatively, modification or damage to a peptide in one part of the protein may lead to a conformation change that leads to elevated trypsin cleavage of otherwise occluded sites in the protein to produce a corresponding increase in the amount of a corresponding undamaged peptide. Overall, about 75–95% of the peptides with increases or decreases in abundance were unmodified in each *E. coli* strain tested (Table 1, Table 2 and Table 3). Far fewer peptides changed in abundance in *D. radiodurans*, suggestive of the powerful effect of enhanced ROS amelioration on suppressing oxidative protein damage. However, of those few peptides, a larger fraction were oxidized (nearly 50%) (Figure 1, Table 4).

The oxidation events that occurred on peptides that were modified and also increased or decreased in abundance are listed in Table 6 and Table 7. The peptides of most interest are those that both exhibit an increase in abundance and are oxidatively modified. We associate significant increases in the abundance (fold change greater than two) of oxidatively modified peptides with the primary effect of irradiation. The total number of modified peptides in this class increased somewhat from our previous study, although perhaps not as much as might be expected due to the 3X increased IR dose used in this study. The overall fraction of the peptides increasing in abundance that were oxidized varied among the four samples. This number ranged from 40% in wild type *E. coli* to 18% and 17% in the evolved isolates of *E. coli* to 78% for *D. radiodurans*. Note that only 17 peptides fall into this category in *Deinococcus*. Again, hydroxylation, especially hydroxylation of methionine, was the most common oxidation event observed (Table 6).

We further investigated the level of oxidation on peptides that appeared only in the irradiated sample. Although such peptides could not be assigned a quantitative fold increase, an oxidized peptide that appeared only in irradiated cells likely represents the most significant form of IR-induced protein oxidation. Nearly 30% of peptides in this category were oxidized in all organisms tested (Figure 1). Again, the most common oxidation event, by a considerable margin, was hydroxylation. Carbonylation was second, followed by rarer deoxidation and trioxidation events. This was true in all samples.

Overall, there were 249 peptides from WT *E. coli* that were both oxidized and present at increased abundance (fold increase greater than two). This number declined to 106 and 65 for IR9-150-1 and IR9-150-2, respectively, suggesting the possible presence of increased ROS amelioration that is adding proteomic protection in these cells. In *D. radiodurans*, 50 peptides with oxidative modifications and increased abundance were detected. Thus, the result with IR9-150-2 suggests that if amelioration of proteome oxidation is present, it may be comparable to that seen in *D. radiodurans*. The total number of proteins represented among these oxidized peptides was 157 for WT *E. coli*, 81 for IR9-150-1, 44 IR9-150-2, and only 38 for *D. radiodurans*.

**Figure 2:**
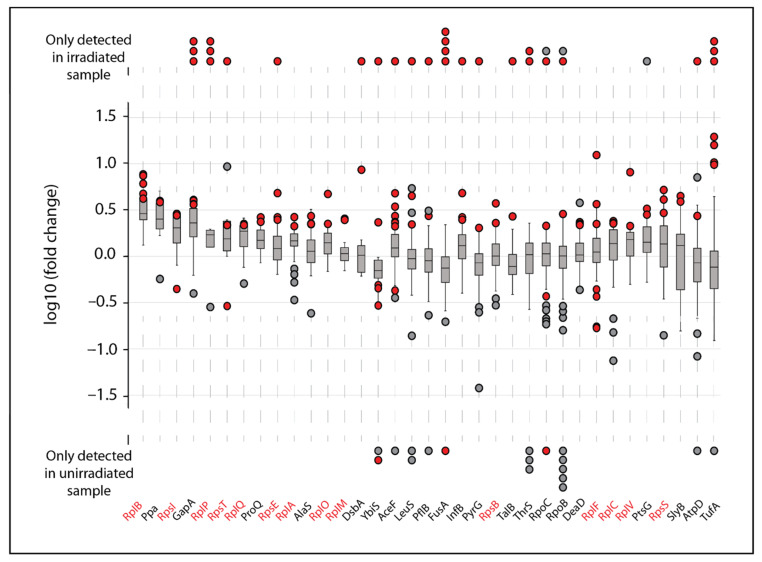
Most oxidation-sensitive proteins in *E. coli* MG1655. Thirty-five proteins with at least two oxidized peptides with increased abundance due to irradiation with 3 kGy were classified as the most IR-sensitive proteins in MG1655, and are listed. The distribution of fold change of all peptides belonging to each protein are depicted as box and whisker plots. Red dots indicate the fold change of oxidized peptides belonging to each protein; red and gray dots indicate oxidized or unmodified peptides, respectively, detected only in the 3 kGy treated (top) or untreated (bottom) samples, and therefore have no quantifiable fold change. Some proteins, such as those on the far left, have nearly all peptides increasing in abundance, suggesting that IR-induced conformational changes may be enhancing mass spectrometry (MS)-based sequencing of the protein. Proteins shown in red are ribosomal proteins.

While such rampant oxidation appears scattershot, we sought to determine what proteins (if any) were more susceptible to IR induced damage than others. We narrowed our analysis to any proteins with at least two oxidized peptides with increased abundance in the irradiated samples (quantified fold change or present in the irradiated sample only). Of the 157 total proteins from the MG1655 dataset that had modified peptides that increased in abundance, only 35 had multiple peptides with modifications (Figure 2). In some cases, as in the four proteins at the far left, all of the peptides detected in both unirradiated and irradiated samples exhibited a similar increase in abundance in the irradiated samples. This suggests that the protein itself increased in abundance during the irradiation. Much more commonly, most of the peptides derived from a particular protein exhibit no change in abundance. Most of these peptides are unmodified, whereas a few peptides from the same protein exhibit increases in abundance. Most of the latter peptides were modified. These are generally proteins that did not change in abundance during the irradiation process, although some of the peptides from those proteins were modified and those modified versions of the peptides exhibited an increase in the irradiated samples. Figure 2 focuses on proteins where multiple modifications were detected, but most of the modified proteins (517 total) had no peptides that increased in abundance (394 such proteins), or only one modified peptide that increased in abundance (123 proteins).

**Figure 3:**
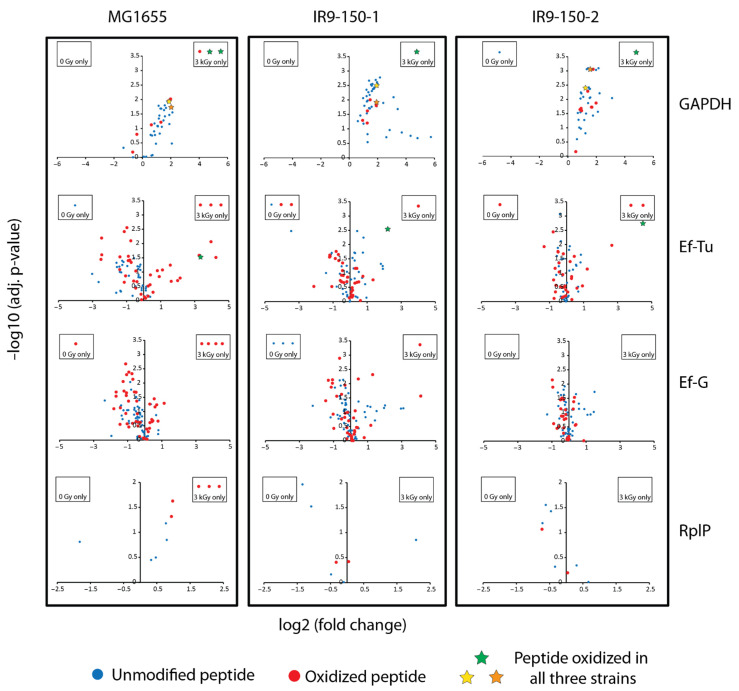
Oxidation of glyceraldehyde 3-phosphate dehydrogenase (GAPDH), elongation factor (Ef)-Tu, Ef-G, and RplP across all three *E. coli* isolates. Volcano plots depict the fold increase or decrease (log2 transformed) and statistical significance of the fold change (−log10 transformed Benjamini–Hochburg adjusted *p*-value). Peptides that were only detected in the 3 kGy irradiated samples are shown in a separate box associated with the appropriate volcano plot. Each dot represents a single peptide, where unmodified peptides are blue, oxidized peptides are red, and modified peptides with a significant fold increase (fold change greater than 2, adjusted *p*-value less than 0.05) detected in all three *E. coli* isolates are depicted as stars. The color of the star denotes the same peptide across the three isolates. For GAPDH, a green star indicates an oxidized active site peptide (YAGQDIVSNASCTTNCLAPLAK), and orange and yellow stars indicate two other oxidized proteins elsewhere in GAPDH (DNTPMFVK and LTGMAFR). For Ef-Tu, the green star indicates an oxidation on the peptide AFDQIDNAPEEK.

Of the proteins displayed in Figure 2, four have multiple oxidized peptides that appeared only in the irradiated samples: GAPDH, elongation factor (Ef)-Tu (TufA), Ef-G (FusA), and the ribosomal protein RplP. These four proteins may represent the most prominent targets of IR-induced protein oxidation. Further analysis revealed that GAPDH, Ef-Tu, and Ef-G are also clear targets of IR-induced oxidation in IR9-150-1 and IR9-150-2 (Figure 3).

We have previously noted the IR-sensitivity of GAPDH, Ef-Tu, and Ef-G [16], and the presence of increased oxidation on these proteins in even IR-resistant *E. coli* isolates highlights their susceptibility.

In particular, only GAPDH and Ef-Tu have modified peptides (three in GAPDH and one in Ef-Tu) that increased in abundance greater than two fold in all three *E. coli* strains (Figure 3, denoted by stars). For GAPDH, one of these peptides was derived from the active site and containing the active site Cys residue, which is subject to trioxidation [16]. In the current study, this GAPDH oxidation event could only be localized to the active site Cys in MG1655, but the mass shifts for the oxidized active site peptide in the evolved isolates are consistent with oxidation of Cys to sulfonic acid (+48 Da). Incomplete oxidation (dioxidation, +32 Da) is also apparent (Supplemental Tables S1–S3). The sequence of this active site peptide is highly conserved in bacteria and much of it is conserved in other organisms. This same peptide does not suffer detectable oxidation in the GAPDH from *D. radiodurans*, even at 3000 Gy. In this gene, *D. radiodurans* (and the rest of the Deinococcus-Thermus phylum) has a rare sequence alteration that eliminates a second Cys residue that is part of the normal active site consensus for this enzyme [43], a change that may render it much less sensitive to oxidation. It has been previously suggested that the ROS-sensitivity of GAPDH may act as a metabolic switch necessary to shift carbon flux to the pentose phosphate pathway to generate NADPH used in reducing glutathione and in biosynthesis [43,44,45,46]. Two more modified peptides from GAPDH were increased in abundance in all three *E. coli* isolates. These peptides map to elsewhere in the protein, and oxidation is likely localized to l-met residues (M129 and M229) in all three isolates (localization to l-met is confirmed in IR9-150-1 and IR9-150-2, but inconclusive in MG1655).

Less evident in earlier work has been the effects of IR on Ef-Tu. A residue of Ef-Tu, F47, may be particularly ROS-sensitive as revealed in this new dataset. This residue is quite close to the GDP binding site. An IR-induced and very prominent hydroxylation event was localized to F47 in a peptide from IR9-150-2 (22-fold increase). Mass shifts corresponding to hydroxylation were detected in the same peptide at increased levels in MG1655 (10-fold increase) and IR9-150-1 (4-fold increase) (Figure 3; see also Figure S1), although the modified residue could not be unambiguously identified in the latter two strains. To our knowledge, oxidation sensitivity of this residue has not been previously reported and the biological consequence of such modification is unknown.

In *Deinococcus radiodurans*, only one protein had multiple peptides that both were oxidized and increased in abundance. This is an SLH protein, part of the S layer on the cell surface, which has three oxidized peptides that increase in abundance. These oxidized peptides were detected only in the irradiated sample. Another protein that is part of the S layer, SlpA, provided one peptide that was the only measurable target of IR-mediated oxidation in our previous trial carried out at 1000 Gy [16]. SlpA also has one oxidized peptide in the current dataset, although the increase in abundance of that peptide is not as great as the three peptides from the SLH protein. *Deinococcus* may not be able to protect cell surface proteins from IR-mediated oxidation as well as proteins in the cell interior. The prominent oxidation events in *Deinococcus* are summarized in Table 8. The entire dataset is provided in Supplemental Table S4. The peptide from SlpA is not included as the increase in abundance is modest.

## 4. Discussion

The work presented in this study leads to multiple conclusions. First, of the oxidation events detected, hydroxylation is the most prominent. Many previous studies have focused efforts on detecting carbonylation and this has been reported to be the main oxidation event affecting proteins [14,47,48]. This and our previous work [16] indicates that much more attention should be focused on hydroxylation events. Second, the overall level of proteomic oxidation is not extensive, even at 3000 Gy. There were only 480 modified peptides from all four samples that exhibited an increase in abundance of > 2-fold. Even in WT *E. coli*, only 157 proteins, out of 2020 detected, exhibited any detectable modification that was clearly attributable to IR. Of these, only 35 had multiple peptides that were so modified. Finally, glyceraldehyde phosphate dehydrogenase again figures prominently in the modification dataset. The new dataset also provided a closer look at Ef-Tu as a major oxidation target.

The proteins that have only one or a few modified peptides that increase in abundance vary from one *E. coli* strain to another but are always few in number. The observed increases are also often small. This may indicate that even with an IR dose of 3000 Gy, we are far from saturation relative to the proteomic oxidative events that might occur. In wild type *E. coli*, the proteome is remarkably resistant to oxidation as long as cells remain intact [16]. The higher IR doses in the present study highlight the presence of some proteins where there are oxidatively modified peptides that increase substantially in abundance while other peptides in the same protein are not modified and do not change in abundance. This suggests that the limited protein oxidation that does occur may be greatly affected by factors such as protein structure and exposure to water, as has also been seen in studies of IR effects on individual proteins such as lysozyme [11].

The presence of a few highly sensitive targets of oxidation appearing in all *E. coli* samples, such as the active site peptide of glyceraldehyde 3-phosphate dehydrogenase and one peptide of Ef-Tu, suggest that proteome oxidation is not at all random. There may be an evolutionary component to protein oxidation potential. Targeted oxidation of these particular peptides in GAPDH and Ef-Tu may improve survival under conditions of oxidative stress. Inactivation of GAPDH directly slows glycolysis, funneling hexose metabolism into the pentose phosphate pathway. The resulting production of NADPH can play a role in ROS amelioration, providing reduced glutathione for glutathione peroxidase [49,50]. The active site of GAPDH is widely conserved, suggesting that an ROS amelioration mechanism of this kind is present in cells from bacteria to humans. Inactivation or partial inactivation of Ef-Tu may also be an adaptive response that improves survival to oxidative damage. Protein biosynthesis typically consumes 80–90% of the chemical energy resources of a cell (ATP; [51]). In principle, a transient slowdown of this process could free resources to deal with DNA damage as well as damage to Fe-sulfur centers and other cellular components. In principle, lower translation rates might also preserve pools of intracellular GTP for repairing oxidative damage to DNA.

The study of proteome oxidation is still in its infancy. As with many of the fields represented in this Special Issue of *Cells*, Miroslav Radman has made early and prominent contributions, pointing out the potential importance of these processes to human health and aging [11,12,13,14,15]. It is a privilege to dedicate this article to Miro on this auspicious occasion.

## Figures and Tables

**Table 1:** Observed peptide oxidation for MG1655 (3 kGy:0 Gy) bacterial strain.

	Total Peptides	Quantified Peptides	Unchanging Peptides	FC > 1	FC > 2	FC < 1	FC < 2	In Treated Only	In Untreated Only
Total	21,516	18,733	16,470	910	387	1353	421	276	355
+14 (Carbonylation)	444	378	287	12	4	79	41	9	11
+16 (Hydroxylation)	1457	1223	968	211	141	44	27	50	23
+32 (Dioxidation)	318	240	206	11	10	23	14	30	4
+48 (Trioxidation)	57	41	36	1	1	4	4	4	1
Unmodified	19,684	17,229	15,260	687	235	1282	376	192	327
Fraction carbonylated	2.2%	2.0%	2.1%	0.8%	1.0%	2.1%	3.8%	4.7%	2.9%
Fraction hydroxylated	7.3%	6.5%	7.1%	13.5%	35.6%	1.2%	2.5%	26.3%	6.0%
Fraction dioxidized	1.6%	1.3%	1.5%	0.7%	2.5%	0.6%	1.3%	15.8%	1.0%
Fraction trioxidized	0.3%	0.2%	0.3%	0.1%	0.3%	0.1%	0.4%	2.1%	0.3%

**Table 2:** Observed peptide oxidation for IR9-150-1 (3 kGy:0 Gy) bacterial strain.

	Total Peptides	Quantified Peptides	Unchanging Peptides	FC > 1	FC > 2	FC < 1	FC < 2	In Treated Only	In Untreated Only
Total	19,867	18,860	13,544	1563	396	3753	1068	190	383
+14 (Carbonylation)	383	361	255	26	6	80	23	4	14
+16 (Hydroxylation)	1341	1233	951	175	64	107	45	24	38
+32 (Dioxidation)	231	206	158	13	4	35	10	4	15
+48 (Trioxidation)	41	37	29	2	0	6	1	0	3
Unmodified	18,254	17,384	12,406	1373	328	3605	1012	162	327
Fraction carbonylated	1.9%	1.9%	1.9%	1.7%	1.5%	2.1%	2.2%	2.1%	3.7%
Fraction hydroxylated	6.7%	6.5%	7.0%	11.2%	16.2%	2.9%	4.2%	12.6%	9.9%
Fraction dioxidized	1.2%	1.1%	1.2%	0.8%	1.0%	0.9%	0.9%	2.1%	3.9%
Fraction trioxidized	0.2%	0.2%	0.2%	0.1%	0.0%	0.2%	0.1%	0.0%	0.8%

**Table 3:** Observed peptide oxidation for IR9-150-2 (3 kGy:0 Gy) bacterial strain.

	Total Peptides	Quantified Peptides	Unchanging Peptides	FC > 1	FC > 2	FC < 1	FC < 2	In Treated Only	In Untreated Only
Total	14,738	13,356	9788	1989	213	1579	66	88	68
+14 (Carbonylation)	194	156	126	17	2	13	5	2	2
+16 (Hydroxylation)	1756	1564	1323	122	34	119	22	14	16
+32 (Dioxidation)	156	117	101	8	1	8	2	11	1
+48 (Trioxidation)	34	25	22	0	0	3	1	1	0
Unmodified	12,792	11,650	8342	1859	178	1449	41	62	51
Fraction carbonylated	1.0%	0.8%	0.9%	1.1%	0.5%	0.3%	0.5%	1.1%	0.5%
Fraction hydroxylated	8.8%	8.3%	9.8%	7.8%	8.6%	3.2%	2.1%	7.4%	4.2%
Fraction dioxidized	0.8%	0.6%	0.7%	0.5%	0.3%	0.2%	0.2%	5.8%	0.3%
Fraction trioxidized	0.2%	0.1%	0.2%	0.0%	0.0%	0.1%	0.1%	0.5%	0.0%

**Table 4:** Observed peptide oxidation for *D. radiodurans* (3 kGy:0 Gy) bacterial strain.

	Total Peptides	Quantified Peptides	Unchanging Peptides	FC > 1	FC > 2	FC < 1	FC < 2	In Treated Only	In Untreated Only
Total	14,440	12,963	12,858	34	17	71	28	95	114
+14 (Carbonylation)	285	225	225	0	0	0	0	5	3
+16 (Hydroxylation)	1367	1159	1145	12	8	2	0	22	18
+32 (Dioxidation)	192	138	132	5	5	1	0	10	4
+48 (Trioxidation)	29	23	22	0	0	1	0	0	0
Unmodified	12,852	11,643	11,559	17	4	67	28	63	92
Fraction carbonylated	1.4%	1.2%	1.7%	0.0%	0.0%	0.0%	0.0%	2.6%	0.8%
Fraction hydroxylated	6.9%	6.1%	8.5%	0.8%	2.0%	0.1%	0.0%	11.6%	4.7%
Fraction dioxidized	1.0%	0.7%	1.0%	0.3%	1.3%	0.0%	0.0%	5.3%	1.0%
Fraction trioxidized	0.1%	0.1%	0.2%	0.0%	0.0%	0.0%	0.0%	0.0%	0.0%

**Table 5:** Amino acid modifications on peptides exhibiting no change in abundance.

	Carbonylation (+14 Da)				Hydroxylation (+16 Da)				Dioxidation (+32 Da)				Trioxidation (+48 Da)			
	MG1655	IR9-150-1	IR9-150-2	*D. radiodurans*	MG1655	IR9-150-1	IR9-150-2	*D. radiodurans*	MG1655	IR9-150-1	IR9-150-2	*D. radiodurans*	MG1655	IR9-150-1	IR9-150-2	*D. radiodurans*
**A**	11	19	20	21	2	1	1	4	16	8	15	18	0	0	0	0
**C**	0	1	2	0	5	2	4	0	4	0	3	4	12	6	16	15
**D**	20	11	10	6	22	23	11	18	8	12	7	6	1	0	0	0
**E**	10	6	1	4	32	24	13	7	15	5	8	4	0	0	0	0
**F**	3	1	3	5	10	14	11	8	4	6	2	3	6	6	8	3
**G**	0	0	0	0	0	0	0	0	0	0	0	0	0	0	0	0
**H**	1	0	1	1	12	9	5	5	0	2	0	3	0	0	0	0
**I**	8	11	5	8	23	23	26	12	6	11	9	5	0	0	0	0
**K**	2	0	0	4	0	0	0	0	1	0	1	0	0	0	0	0
**L**	9	9	12	21	53	35	40	52	20	6	7	16	0	1	0	1
**M**	11	5	7	10	388	308	537	392	18	9	20	21	0	0	0	0
**N**	7	5	9	6	26	13	15	18	6	2	6	2	0	0	0	0
**P**	5	8	6	12	27	18	15	32	15	8	14	21	1	0	0	1
**Q**	9	8	2	0	23	12	5	11	1	2	1	0	0	0	0	0
**R**	9	1	2	2	8	7	3	5	7	3	1	3	0	1	0	0
**S**	13	14	11	8	22	22	18	13	9	7	5	9	1	0	0	0
**T**	21	16	11	9	26	22	26	26	10	9	14	11	0	1	0	0
**V**	43	31	28	32	46	33	33	29	13	17	15	15	3	0	0	0
**W**	11	10	0	2	50	54	15	19	95	55	11	16	24	18	2	1
**Y**	181	149	49	77	25	11	3	10	11	13	2	5	13	7	6	9
**Total**	**374**	**305**	**179**	**228**	**800**	**631**	**781**	**661**	**259**	**175**	**141**	**162**	**61**	**40**	**32**	**30**

Highlighting is used to draw attention to the most common types of amino acid modifications. Darker shading = more common.

**Table 6:** Amino acid modifications on peptides exhibiting an increase in abundance.

	Carbonylation (+14 Da)				Hydroxylation (+16 Da)				Dioxidation (+32 Da)				Trioxidation (+48 Da)			
	MG1655	IR9-150-1	IR9-150-2	*D. radiodurans*	MG1655	IR9-150-1	IR9-150-2	*D. radiodurans*	MG1655	IR9-150-1	IR9-150-2	*D. radiodurans*	MG1655	IR9-150-1	IR9-150-2	*D. radiodurans*
**A**	0	1	1	1	0	0	0	0	5	1	2	0	0	0	0	0
**C**	0	0	0	0	2	0	0	0	3	0	0	0	5	0	0	0
**D**	1	0	1	0	3	0	0	1	2	0	0	0	1	0	0	0
**E**	0	0	0	0	4	2	2	0	2	0	0	0	0	0	0	0
**F**	1	0	0	0	3	3	2	2	2	0	0	0	1	0	1	0
**G**	0	0	0	0	0	0	0	0	0	0	0	0	0	0	0	0
**H**	0	0	0	0	4	2	0	0	0	0	0	0	0	0	0	0
**I**	1	0	0	0	2	1	0	0	0	0	0	0	0	0	0	0
**K**	0	0	0	0	0	0	0	0	0	0	0	0	0	0	0	0
**L**	0	1	0	0	3	4	1	0	3	0	0	0	0	0	0	0
**M**	0	0	0	2	100	29	25	8	2	0	0	3	0	0	0	0
**N**	1	0	0	0	0	0	1	1	1	1	0	0	0	0	0	0
**P**	0	2	0	0	2	2	0	2	0	1	1	1	0	0	0	0
**Q**	0	0	0	0	5	0	0	1	0	0	0	0	0	0	0	0
**R**	0	0	0	0	0	0	0	0	1	0	0	0	0	0	0	0
**S**	0	3	0	1	2	1	0	1	1	1	0	2	0	0	0	0
**T**	0	1	0	0	2	1	0	1	1	0	0	1	0	0	0	0
**V**	1	0	2	0	11	2	0	3	1	0	1	0	0	0	0	0
**W**	1	1	0	0	5	1	2	1	21	1	4	5	0	0	0	0
**Y**	6	1	1	0	0	1	0	1	0	1	0	1	0	0	0	0
**Total**	**12**	**10**	**5**	**4**	**148**	**49**	**33**	**22**	**45**	**6**	**8**	**13**	**7**	**0**	**1**	**0**

Highlighting is used to draw attention to the most common types of amino acid modifications. Darker shading = more common.

**Table 7:** Amino acid modifications on peptides exhibiting a decrease in abundance.

	Carbonylation (+14 Da)				Hydroxylation (+16 Da)				Dioxidation (+32 Da)				Trioxidation (+48 Da)			
	MG1655	IR9-150-1	IR9-150-2	*D. radiodurans*	MG1655	IR9-150-1	IR9-150-2	*D. radiodurans*	MG1655	IR9-150-1	IR9-150-2	*D. radiodurans*	MG1655	IR9-150-1	IR9-150-2	*D. radiodurans*
**A**	1	1	0	0	0	0	0	0	0	1	1	1	0	0	0	0
**C**	0	0	0	0	2	0	0	0	0	0	0	0	1	2	0	0
**D**	10	0	1	0	0	3	1	1	0	1	0	0	0	0	0	0
**E**	2	0	1	0	3	2	0	1	3	0	0	0	0	0	0	0
**F**	1	0	0	1	1	0	0	1	1	1	0	0	0	1	1	0
**G**	0	0	0	0	0	0	0	0	0	0	0	0	0	0	0	0
**H**	0	0	0	0	1	2	1	0	0	1	0	0	0	0	0	0
**I**	0	5	0	0	1	5	1	0	0	2	0	0	0	0	0	0
**K**	0	0	0	0	0	0	0	0	1	0	1	0	0	0	0	0
**L**	0	0	0	0	3	4	0	1	3	1	0	0	0	0	0	0
**M**	3	2	0	0	18	20	10	3	4	0	0	1	0	0	0	0
**N**	0	0	0	0	3	0	0	1	0	0	0	0	0	0	0	0
**P**	0	0	0	0	2	2	2	0	1	0	0	0	0	0	0	0
**Q**	0	0	0	0	4	2	0	2	0	0	0	0	0	0	0	0
**R**	1	0	0	0	2	0	0	0	3	0	0	0	0	0	0	0
**S**	1	0	0	0	2	4	1	0	0	0	0	0	0	0	0	0
**T**	4	2	0	0	3	3	0	0	0	1	0	0	0	0	0	0
**V**	6	2	2	1	6	2	3	0	2	2	0	0	0	0	0	0
**W**	3	1	0	0	7	7	1	1	3	11	0	0	3	1	0	0
**Y**	38	17	2	0	3	1	0	0	2	1	0	0	3	0	0	0
**Total**	**70**	**30**	**6**	**2**	**61**	**57**	**61**	**11**	**23**	**22**	**2**	**2**	**7**	**4**	**1**	**0**

Highlighting is used to draw attention to the most common types of amino acid modifications. Darker shading = more common.

**Table 8:** Prominent oxidation events caused by irradiation at 3000 Gy in *Deinococcus radiodurans*.

Annotated Sequence	Modifications	Master Protein Accessions	Master Protein Descriptions	Gene	Abundance Ratio: (3 kGy)/ (0 kGy)	Abundance Ratio Adj. *p*-Value: (3 kGy)/(0 kGy)
[R].MYVDKGMSWADSASLQAIR.[S]	2× Oxidation [M1; M7]	Q9RTJ4	Uncharacterized protein	DR_1768	15.06	0.023576883
[R].LGDLNDTEKQWASLSAAK.[L]	1× Dioxidation [W11]; 2× Oxidation [Q10; S13]	Q9RTJ4	Uncharacterized protein	DR_1768	5.118	0.037732817
[R].INGMASGTANQDVTALTAR.[I]	1× Dioxidation [M4]	Q9RVA5	SLH family protein	DR_1124	1000 only in IR sample	
[R].MLSTNALSTCGLSQGDMTVVMNGMQEVSTLAAIATR.[V]	2× Dioxidation [M17; M21]	Q9RVA5	SLH family protein	DR_1124	1000	
[R].IAAGQTNAGYGATTGSATDPYALGLVGVEYR.[V]	1× Oxidation [T/Q/Y]	Q9RVA5	SLH family protein	DR_1124	1000	
[R].LTWDGNQNYDK.[L]	1× Dioxidation [W3]	Q9RZK2	Iron ABC transporter, periplasmic substrate-binding protein	DR_B0125	15.855	0.008996
[K].VVVVAPFAGGNNWVYSNVR.[L]	1× Dioxidation [W13]; 2× Oxidation [W13; Y15]	Q9RZK2	Iron ABC transporter, periplasmic substrate-binding protein	DR_B0125	1000	

## Data Availability

MS datasets for each strain are available online at the Proteomics Identification Database (https://www.ebi.ac.uk/pride/; accession number: PXD024784) and are included as Supplementary Tables S1–S3.

## Supplementary Materials

The following are available online at https://www.mdpi.com/article/10.3390/cells10040954/s1, Figure S1: Example peptide spectra, Supplementary Tables S1–S4: Mass spectrometry data from all four strains.
