# Supplementary material for: Proteome Damage Inflicted by Ionizing Radiation: Advancing a Theme in the Research of Miroslav Radman
Source: Cells. 2021 Apr 20;10(4):954. doi: 10.3390/cells10040954 (PMC8074248; doi:10.3390/cells10040954)
Supplement: Supplementary file 1 [file cells-10-00954-s001.zip › Supplemental files/Figure S1.pdf]

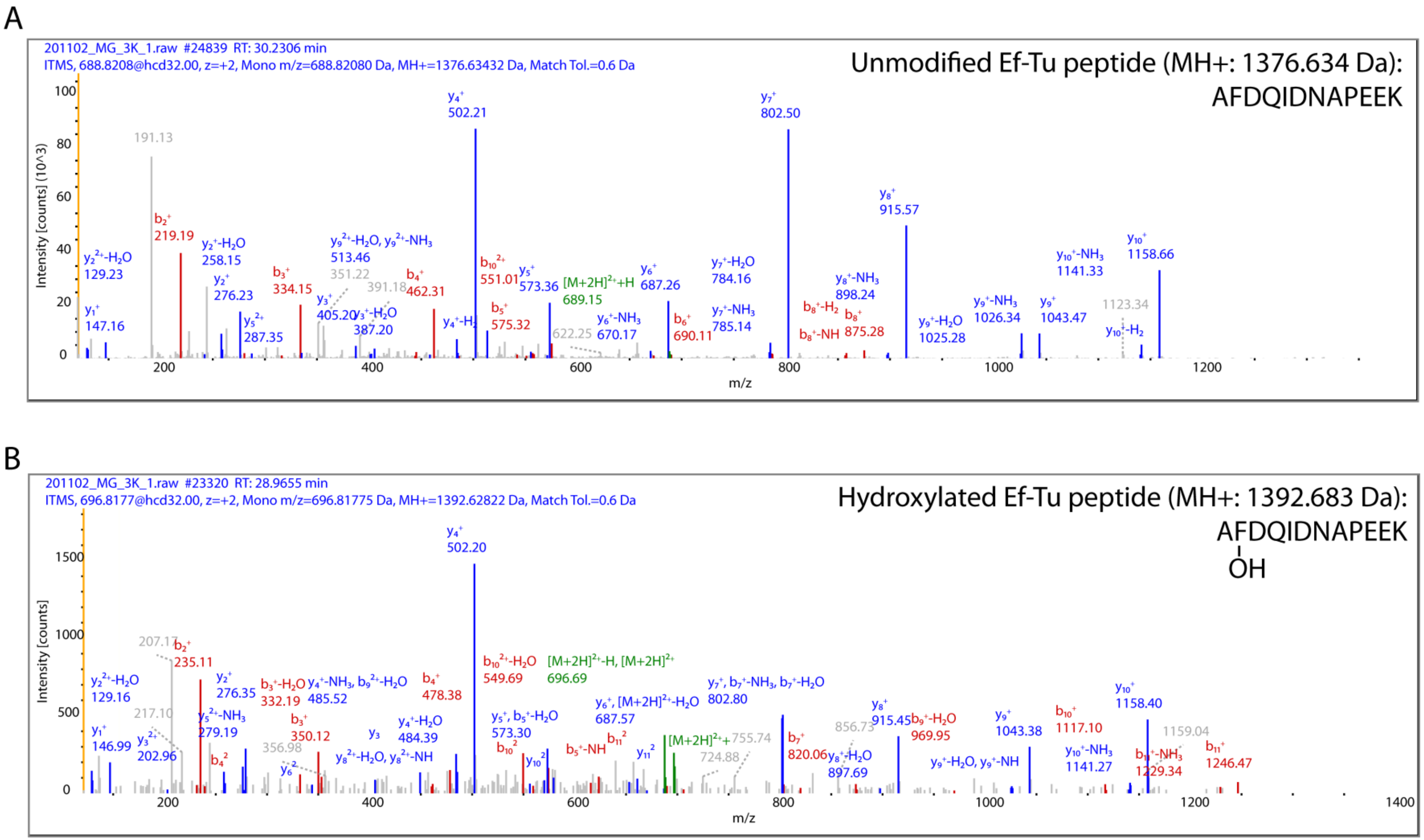

Figure S1. Example peptide spectra. An example A) unmodified and B) hydroxylated peptide from EF-Tu (AFDQIDNAPEEK) is shown. The hydroxylated form of this peptide is increased in abundance in irradiated samples for all three Escherichia coli isolates tested. Y-ion series are shown in blue, b-ion series are shown in red, and precursor ions are labelled with green. Neutral losses per ion are shown as "-X" (- H_2O for water, for example). The ionized peptide mass (MH+) I given to highlight the mass difference between each peptide at z= 2 (+8.0 mass adduct).
